# Understanding the experiences of PHC nurses in caring for older patients in the post-fifth wave of the COVID-19 pandemic: an exploratory qualitative study

**DOI:** 10.3389/fpubh.2024.1340418

**Published:** 2024-04-18

**Authors:** Barbara Ślusarska, Grzegorz Józef Nowicki, Agnieszka Chrzan-Rodak, Ludmiła Marcinowicz

**Affiliations:** ^1^Department of Family and Geriatric Nursing, Medical University of Lublin, Lublin, Poland; ^2^Department of Obstetrics, Gynaecology, and Maternity Care, Medical University of Bialystok, Bialystok, Poland

**Keywords:** qualitative research, experiences, primary health care, nurses, older adult, COVID-19, pandemic, health services for the aged

## Abstract

**Objective:**

To ensure the best possible care, the perspective of PHC nurse work experience during the COVID-19 pandemic should be considered when developing nursing care protocols for older patients who receive PHC services.

**Method:**

This exploratory qualitative study was conducted with 18 nurses working continuously in PHC between the first and fifth waves of the pandemic. Semi-structured thematic interviews were undertaken. Qualitative thematic content analysis was conducted to identify and group the themes that emerged from the discourse. Interviews were transcribed and analyzed using thematic analysis.

**Results:**

The first topic describes the nurses’ experiences of physical and mental suffering in caring for older patients in response to the pandemic. The second topic covers the experience of reorganizing PHC work. The third topic focuses on the difficulties of caring for older patients. The final topic includes issues of support needs for nurses in PHC work.

**Conclusion:**

The experience and understanding of PHC nurses in caring for older people during the COVID pandemic should lead to significant changes in the system of nursing care for geriatric patients and in the cooperative role within geriatric care specialist teams. Drawing on the experience of COVID-19, it is necessary to work on the weak points of PHC exposed by the pandemic in order to improve the quality of care and life for geriatric patients.

## 1 Introduction

Pandemic coronavirus disease 2019 (COVID-19) has caused significant mortality worldwide. When the COVID-19 pandemic was compared across Europe in terms of numbers of deaths, crude rates, and adjusted mortality trend ratios (AMTRs), it was found that Eastern European countries (Hungary, the Czech Republic, Slovakia, and Poland) had higher AMTRs than Western European countries (1). This outcome is related to social, economic, and healthcare quality factors (2).

The SARS-CoV-2 pandemic has led to an alarming crisis in healthcare systems, including Primary Health Care (PHC) worldwide, creating unique challenges in regard to the care of older patients with special needs and routine medical care (3, 4) - with the older patients being the most disadvantaged. One of the main guidelines for the care of older people during the COVID-19 pandemic in home and primary health care settings is the proactive assessment by a primary care physician or nurse to identify early signs of COVID or abnormal symptoms like sudden changes in cognition, onset of behavioral disturbances, or functional deterioration that may indicate early signs of the infection (5). As a result of the above conditions, geriatric care professionals, including nursing care, have been forced to adopt new ways of working quickly to ensure continuity of care for older patients in response to the COVID-19 pandemic (6).

The primary healthcare sector played and continues to play an essential role in the pandemic response. During the COVID pandemic-19, in addition to taking steps to ensure the continued availability of primary care services, physicians and nurses were responsible for operationalizing public health priorities (such as surveillance and vaccination) as well as ensuring that facilities can respond to the growing demand for acute care services, and helping to maintain continuity of care for people with chronic diseases, as well as for older patients (7–9). The role of PHC facilities during the COVID pandemic-19 was mainly focused on identifying, managing and monitoring COVID-19 cases and their treatment. In addition, they played a key role in the rapid implementation of mass vaccination and thus in influencing the reduction of demand for hospital services (10, 11).

Caring for patients with COVID-19 requires better professional competence from care providers based on new knowledge and training (12). However, reports by Liu et al. (13) confirm that the nurses provided this care without adequate training. Nurses, therefore, needed additional training and ongoing educational support to improve their preparedness and the effectiveness of care they provide in times of crisis, as well as to better manage mental burden issues and manage well-being (14).

Limited research exists regarding nurses’ perspectives and experiences in caring for patients with COVID-19 (15). Some focuses mainly on the study of the physical and psychological suffering of nurses while caring for COVID patients (10) or the challenges nurses have faced in providing care, e.g., stress, feeling ineffective, fatigue and difficulties in using personal protective equipment while at work (16). Much of the other research, the available qualitative research on the experiences of healthcare workers (including nurses) during the COVID-19 pandemic, focuses mainly on specialist care in the hospital (17–19).

PHC is often the first link to which a geriatric patient in need of medical care is exposed to. Non-standard situations such as the COVID-19 pandemic reveal the shortcomings of this system. The qualitative research conducted allowed us to recognize the range of problems that nurses and patients have to struggle with, not only in times of crisis, but often also on a daily basis. Understanding the experiences of nurses in caring for older patients, in the context of caring for people who remain at home using PHC services, helps in learning about these conditions, and, based on them, enables the formulation of protocols for safe and reactive care with regard to the changing needs of older patients in the face of the COVID pandemic and other epidemic or crisis situations.

Therefore, further exploration of the experiences of this group of nurses is needed, especially a perspective based on the experience of geriatric care toward older patients who had special needs during the COVID pandemic-19. Thus, the present study was designed to investigate the experiences of PHC nurses who cared for older patients in the PHC during the SARS-CoV-2 infection pandemic in Poland.

## 2 Materials and methods

### 2.1 Study design

This exploratory qualitative study used individual, semi-structured interviews to understand the experiences of PHC nurses in caring for older patients in the post-fifth wave of the COVID-19 pandemic. The study is presented in line with the “Consolidated criteria for reporting qualitative studies (COREQ) guidelines” (20).

The participants were nurses who cared for older patients during the pandemic of SARS-CoV-2 infection, in primary care, in a city located in south-eastern Poland with a population of more than 300,000. A targeted sample of nurses was recruited from selected PHC facilities, and then after obtaining permission from the institution’s managers, a snowball sampling method was employed to recruit potential participants (21, 22). The inclusion criteria were as follows: (1) nurses who cared for older patients during the entire period [from the first to fifth wave (23)] of the COVID-19 pandemic in state primary care facilities; (2) those who spoke Polish; and those (3) who volunteered to participate in the study and were able to talk about their experiences. The exclusion criteria included: (1) nurses working in the private PHC sector (2) no consent to participate in the study. Participants were selected with maximum diversity in terms of education, age, length of service and locations of PHC services.

### 2.2 Data collection procedure

In-depth face-to-face free-form interviews were conducted between August and September 2022 by two female researchers, using an interview guide developed by the research team based on the thematic literature review and the purpose of this research (Table 1).

**Table 1:** Interview guide.

Interview guide	
1.	Can you tell me about your experience as a PHC nurse in caring for older patients during the social isolation restrictions of the COVID-19 pandemic?
2.	What emotions did you experience in the workplace during the corona virus pandemic?
3.	Can you tell me about your experience with the tele-treatment given to older patients and their families during the pandemic?
4.	Can you tell me about your experience providing direct care to an older patient with COVID-19 in-home care?
5.	What kind of support did you receive to provide adequate care for older patients during COVID-19?
6.	What barriers and changes were present in your experience of providing older patient care in the PHC during periods of heightened restrictions related to the COVID pandemic-19?
7.	Are there other older patient care experiences from during the COVID pandemic-19 that are important today for providing the best possible care to seniors in home care settings?

After clarifying the objectives of the study and obtaining written informed consent from the participants, an interview date convenient for the participant was established. The interviewers held master’s degrees in nursing with experience in qualitative interviewing, had worked as nurses with older patients, and had experience in COVID-19 prevention and control. They were free to adapt the questions within these topics depending on the situation. The interviewers and participants did not have any personal connection or relationship prior to the interviews. If new themes emerged from the interviews, they were added to the list of topics and used in subsequent interviews. When data saturation was suspected, three additional interviews were conducted to see if they yielded additional information. Data saturation was assumed if no additional information was obtained, and no further interviews were conducted.

Individual interviews were held in a separate room in peaceful and quiet conditions, without interruptions, and interviewees were allowed to withdraw consent at any time. As a result, interviewers remained neutral in data collection and a friendly, comfortable atmosphere was established. The interviews were recorded on a digital medium of audio files after obtaining written informed consent from the participants. The duration of the interviews ranged from 45 min to 60 min.

### 2.3 Data analysis

Data was analyzed using thematic analysis (24) to identify patterns in the data and major themes that represent the participants’ experiences. Within 24 h of each interview, the recording was transcribed. The recordings were first transcribed verbatim and labeled with an identification number. Subsequently, all participant personal data was removed from the transcript. In the next stage, the study participants were given the transcripts to check for accuracy and consistency with their experiences (25). Two researchers reviewed the material independently from the corroborated transcripts, summarized and extracted significant statements, and identified the relevant themes and subthemes, creating a thematic map. Finally, contradictory opinions on the content of the topic were discussed and resolved by a study group consisting of a researcher with a master’s degree in nursing, a doctorate in nursing, a professor of health sciences and a PHC practice nurse - the PHC head nurse, to be exact. When writing the detailed description, two researchers translated the relevant quotations into English from Polish, and a consensus was reached.

### 2.4 Trustworthiness of the study

To ensure the rigour of qualitative research, the four-dimensional criteria of credibility, dependability, confirmability and transferability, according to Lincoln and Guby (26), were applied. Once the initial identification codes were assigned, the participants’ views were verified for accuracy within the range of transcripts. Two academics and qualitative research experts applied a control method to verify the data’s validity. Once the selected codes were established, the content of the transcripts was divided into themes and subthemes.

To achieve credibility, i.e., to be sure that the results from the participants’ perspective are true and reliable, prior to the actual interview sessions, the protocol was tested via two introductory meetings and by using two pilot interviews. In addition, participants were given a copy of the transcripts to check for consistency. Continuous observation of the data was ensured by identifying themes and subthemes, including reading and rereading the data and identifying statements and meanings related to the nurses’ experiences. In addition, participants were given a summary report based on the findings to ascertain whether the analysis truly reflected their experiences.

Dependability was achieved by applying accepted standards, i.e., consolidated criteria for reporting qualitative studies (COREQ) guidelines, as well as by using thematic analysis.

To ensure confirmability, i.e., to increase confidence that other researchers would confirm the results, the first researcher identified potential statements and meanings within the initial transcript, and these meanings were discussed with other researchers to obtain consensus. In addition, there was an ongoing discussion concerning any new meanings. Finally, the first and second researchers categorized meanings and identified themes and subthemes. The process was completed when consensus was reached among the team members on the discovered themes.

Transferability was ensured through a combination of purposive sampling techniques and quantified operational data saturation via the selection of contextual information (location, sample, sample size, sampling strategy, socio-demographics, inclusion and exclusion criteria, and interview procedure).

### 2.5 Ethical considerations

This study was reviewed and approved by the Bioethics Committee of the Medical University of Lublin (Number: KE-0254/73/2021). All participants gave informed written consent after being presented with the purpose, scope, and procedure of the study. Participation in the study was voluntary; at each stage, the participant had the right to withdraw from the study.

## 3 Results

### 3.1 Demographic characteristics

Qualitative interviews were conducted with 18 nurses working in PHC. All participants were female (100%), aged between 20 and 59 years, with a mean age of 44.28 years (SD = 11.11). Their length of service in PHC ranged from 1 to 30 years, with an average of 11.11 (SD = 9.18). Five nurses held a Diploma in nursing (27) and five - a Bachelor of Science (BSc) in nursing, and eight - a Master of Science (MSc) in nursing. Specialization in family nursing was held by 12 (66.6%) of the nurses surveyed. Table 2 shows the demographic characteristics of the participants.

**Table 2:** Demographics of participants (*n* = 18).

Characteristics of nurses		Number (%)
Age	20–29 years	3 (16.7)
	30–39 years	3 (16.7)
	40–49 years	4 (22.2)
	50–59 years	8 (44.4)
Gender	Male	0
	Female	18 (100)
Marital status	Single	5 (27,8)
	Married	13 (72.2)
	Widowed/Divorced	0
Place of residence	Village	3 (16.7)
	Town	10 (55.5)
	City	5 (27,8)
Education in nursing	Diploma in Nursing*	5 (27.8)
	BSc in Nursing	5 (27.8)
	MSc in Nursing	8 (44.4)
Years of work experience in primary health care	1–5 years	7 (38.8)
	6–10 years	3 (16.7)
	11–15 years	3 (16.7)
	16–20 years	2 (11.1)
	21–25 years	1 (5.6)
	26–30 years	2 (11.1)
Specialization	Yes	12 (66.6)
	No	6 (33.4)

*Diploma in nursing - Secondary Nursing School (Lyceum) - Nursing education system in Poland before 1999 (27).

### 3.2 Results of the analysis

As a result of the content analysis of the transcripts, four key themes and related subthemes were identified (Table 3). To maintain the anonymity of the participants’ data, identification numbers (IDs 01 to 18) were used in reporting the results.

**Table 3:** Thematic map.

No.	Themes	Sub-themes
1.	Physical and psychological suffering among nurses	- Fear of COVID-19 and the prospect of change over time - Effect on mental health - Negative impact on the family - Stigma and social isolation
2.	Reorganization of work in PHC	- Reduced accessibility to medical care - Fewer face-to-face visits to outpatient clinics in PHCs - Tele-treatments - Ongoing interventions related to COVID-19 disease in PHCs - Restrictive protective measures against transmission of COVID-19 infection
3.	Difficulties in caring for older patients in PHCs	- Concerns about COVID-19 infection during visits and the most common health problems of older patients - Tele-treatment not adapted to the emotional needs of the older patient - Difficult interpersonal contact with older patients during tele-treatments
4.	PHC nurse support and positive change after the COVID pandemic experience	- Information support - Material support - Emotional support - Nurses’ educational needs - Positive changes after COVID pandemic experience in PHCs

### 3.3 Theme 1: physical and psychological distress among nurses

This topic discusses various aspects of the psychological and physical sensations experienced by nurses at work in the PHC during the COVID pandemic-19.

#### 3.3.1 Fear of COVID-19 and the prospect of change over time

The risk of contracting the virus and developing COVID-19 through work and contact with potentially infected people was frightening for the study participants: “(…) *at work during the coronavirus pandemic; I was certainly accompanied by fear and a kind of uncertainty. As time went on, all this diminished, and later, at some stage, a kind of certainty returned at work, and there was less fear*” (ID 02). Nurses also reported manifestations of panic about performing tasks at risk of virus infection: “*Then there was one big panic because we were so looking at each other between friends, which one should go to the appointment (to the patient’s home) because it was like a conviction”*(ID 10), as well as the statement: “*There have been times when they (patients) have come to the PHC’s office for an injection (shot), and then the same patient calls and says they are breathless and can no longer breathe. I will never forget the horror in their voice and those questions about what to do next. With the older persons it was identical horror simply as dyspnoea, fear and panic appeared, which then transferred to us”* (ID 12).

Nurses also perceived an overwhelming fear of the virus in older people: “*The older patients- you do not even know how they were afraid of this corona virus which also affected us”* (ID 09). However, nurses’ experiences changed throughout the pandemic: “*If I were to go now for a home visit to a patient with suspected COVID, I think I would go. It does not impress me much anymore, now we know a bit more about the virus; we have procedures and vaccinations*” (ID 10).

#### 3.3.2 Effect on mental health

The experiences of nurses working in PHC during the pandemic also impacted their psychological mindset in the form of perceived anxiety, tension, restlessness, fatigue, depression or depressive disorders. This fact is evidenced by the statements: “*At one point in this pandemic, I suffered from depression, I was very depressed about it all, I felt constant anxiety and tension. The messages on television were overwhelming”*(ID 16) and: “*Certainly, in the pandemic I was accompanied by fatigue. It was related to such a constant fear for my health (…) I was tired of persisting in this pandemic and in these procedures and restriction”* (ID 17).

In addition, nurses reported physiological discomfort from having to wear personal protective equipment at work: “*I was annoyed that we had to put on masks because I was suffocating terribly in those masks*” (ID 09) or: “*I also felt tired of having to dress in personal protective equipment* (ID 17).

Participants witnessed the suffering of patients but also encountered situations of unexpected deaths among their patients or relatives or acquaintances: “*A friend of mine, for example, died. I just know that people die from it and at our clinic, we have thinned out our patients and specifically and it was the young people who were dying*” (ID 07).

#### 3.3.3 Negative impact on the family (fear of transmission of COVID-19 infection)

Almost all nurses surveyed expressed fear and anxiety related to both their health and the risk of infection of relatives, as cited: *“I felt the pressure of exposure, anxiety about my health, some fear for my family and their health. I was afraid for my health, I definitely washed myself more thoroughly, took off work clothes when I came home and had a bath right away so I would not have contact with my family directly after coming from work”* (ID 06).

Some participants indicated it was difficult for the family members to understand the situation: “*My child at one time was very frightened when it turned out that we had to go into quarantine. Admittedly, we were there for six days, but it was crazy. Then I had to go to work and my daughter even shouted at me - no, do not go there, because you’ll bring something again, because something will happen again*” (ID 07).

Most nurses verbalized fear for the health of their loved ones: “Fear always for the loved ones, never for oneself. For older patients too, but for the loved ones above all” (ID 08). In later experiences, in parallel to fear, there was irritation: “*Fear and anxiety about your health and the health of your loved ones, then it’s perhaps more of an irritation that nothing is getting back to normal and it goes on and on*”(ID 15).

#### 3.3.4 Stigma of distance and social isolation

Nurses felt the effects of social distancing and their patients’ unwillingness to interact with medical staff expressed as follows: *“Medical personnel were at one point treated as if we were transmitting this virus and people did not want to have contact with doctors or nurses”* (ID 14).

Working during the pandemic was associated with a stigma of social isolation, which was a challenging experience for many: *“All in all, on the one hand, I’m glad I was able to go to work normally because I would probably have gone mad at home locked inside four walls*” (ID 16).

### 3.4 Theme 2: PHC work reorganization

This theme highlights the readiness of nurses to provide care under conditions of PHC reorganization during the pandemic and the difficulties involved, but with a sense of professional duty.

#### 3.4.1 Reduced accessibility to medical care

The pandemic caused significant limitations in accessing doctors in the PHC system, and nurses witnessed challenging experiences for older patients: *“The doctor did not accept a medical appointment then (in-person appointment), everything was by tele-treatment. We explained to the patients that an in-person appointment was impossible. The older patient was sometimes barely on their feet, and we said that everything was done by phone, and the patient said, but I came, well, we said you did not have to come. It was just difficult for them to get used to the situation somehow* (ID 15) and: *“Generally there was not so much direct care initially, everything was closed, there was chaos and isolation. I think the older people were looking for more contact with people later on because they felt isolated”* (ID 18).

Nurses also referred to the limitations of making home visits to patients: *“During the pandemic in geriatric care, we were the ones who did not go to patients like that; if there was a need, very occasional outings were for home appointments*” (ID 08), and also: “*Later, when needed, we would go to homes, but mainly to such 65 plus patients, but this was as needed when justified”*(ID 18). They also perceived difficult contact with the patient: “*I felt nervous about this impediment to get in contact, especially with older patients “*(ID 14).

#### 3.4.2 Fewer face-to-face visits to outpatient clinics in PHC facilities

The reorganization of the PHC working system during the pandemic was expressed in the reduced number of inpatient visits and an increase in telephone medical consultations: “*There were different stages of this COVID pandemic because there was a stage where COVID patients were practically not admitted, because there were only telephone medical consultations available. Then it changed. Patients started to come (to the outpatient clinic), they were examined in a separate office, they entered through a different entrance so that they did not come into contact with other patients”* (ID 02).

#### 3.4.3 Telephone medical consultations

Nurses’ experiences of telephone medical consultations in PHC facilities varied, both positive and negative feedback related to good work organization was expressed: “*Telemedicine were rather well organized. We registered patients every 15 min for a tele-treatment. It was the doctor who called (the patient)*” (ID 08).

In some clinics, especially those with a large number of patients, there were difficulties with telephone medical consultations, both for the patients and for nurses: “*We only had two telephones; people were calling, they could not get through. After about two months, they bought the third phone because we could not cope (…) with 11,000 patients and almost all of them were older. We had three nurses on duty at the registration desk and there were phone calls all the time. It was unmanageable. People were calling like crazy and we were barely able to cope*” (ID 09).

Emergencies during telemedicine calls required quick decisions by nurses: “*Someone called with an urgent case such as a high fever or a drop in saturation. Sometimes we helped to decide whether this person should call the emergency room or, for example, we asked the doctor to add the patient to the tele-treatment”* (ID 05).

#### 3.4.4 Ongoing interventions related to COVID-19 disease in the PHC

Nurses also reported the difficulties associated with implementing direct nursing interventions during the COVID pandemic: *“The patients, as we walked around, were in full coveralls. The appointments were quick, with no time to talk, to go in and give an injection or a dressing and leave, because I did not want to infect myself or infect the patient; everyone was scared”* (ID 13).

Home visits made by nurses were infrequent, limited to emergencies only: *“Home visits were very rare. Only in really exceptional situations, for example, when someone had to have a catheter changed or blood taken for tests. In such cases, we took coveralls and masks and tried to protect ourselves. We also took equipment for the patient, a mask in case the patient did not have a mask”* (ID 17).

Participants also highlighted reduced mental health support activities for patients: “*During the interventions, there was no time for any kind of conversation, psychological support (of the patient), because there were such recommendations to stay at home with the patient as briefly as possible. From what I remember, there were guidelines to do the right thing and leave so as not to get infected by the patient”* (ID 04).

#### 3.4.5 Restrictive protective measures against transmission of COVID-19 infection

Restrictive protective measures against the transmission of COVID-19 infection in relation to their work placed a heavy professional burden upon the nurses: “*As it turned out that there was a positive COVID result in a patient, it was usually after every such presumption that it was disinfected with all these agents, irradiated with lamps, these doorknobs and doors, surgeries* etc.*”* (ID 17) or: “*Home visits were implemented with all precautions, protective clothing and protective measures. Any treatment, whether it was blood drawing or dressing, or even there was a situation where a prescription was carried home to the patient in protective clothing*” (ID 07).

Factors associated with the isolation of patients suspected or ill with COVID were attached to nurses’ experiences of the restrictive measures in PHC facilities: “*There was a dedicated office like this and patients with suspected COVID-19 were admitted there with a sanitary regime*” (ID 04) and: *“A patient with COVID could come in, we had an isolation room*” (ID 05).

### 3.5 Theme 3: difficulties in caring for the older patient

This theme encompasses many issues of caring for older patients during the pandemic, including their concerns, health issues, and the range of difficulties related to the distinctiveness of older patients functioning in the context of the new challenges of the epidemic crisis.

#### 3.5.1 Concerns about COVID-19 infection during appointments and the most common health problems of older patients

Nurses encountered concerns expressed by older patients about viral infection: *“When there was a suggestion, for example, that a patient should come in for a consultation, you could say that 70% of patients, however, refused”* (ID 01) or: “*These older patients were very frightened, preferring telemedicine to visiting the PHC in person*” (ID 03), as well as: *“(…) because now they did not leave the house at all and did not even contact their families, so they did not want to visit the PHC either. And when they did go in, they also wore gloves and masks and wanted to leave as quickly as possible”* (ID 17).

Participants also perceived signs of older people being lost in the restrictions of the epidemic: *“Some of the older persons were very scared, some relatively not, they went without masks, they came to the clinic without masks either, because they could not cope with them anymore”* (ID 08).

The main health problems of older patients in the PHC focused on the symptoms of COVID: *“During this pandemic, when it comes to the most common problems for patients, it is mainly shortness of breath, coughing and weakness”* (ID 10). Health problems related to chronic disease management were less important at the time: *“In most cases, patients just reported co-morbidities such as hypertension, diabetes, and reported when they ran out of blood glucose strips or something like that, or to have prescriptions for medicines, but there were also colds, there were very many colds*” (ID 15).

#### 3.5.2 Tele-treatment not adapted to the emotional needs of the older patient

The participants reported their perceptions concerning the deprivation of face-to-face conversation and the support for older patients during tele-treatment previously received during inpatient visits. This is evidenced by statements: *“One doctor, a specialist in geriatrics, has such a good approach to these older people you could say that to a certain extent they have become addicted to her, to these visits. They also really enjoyed talking to our doctor, which is what they missed about tele-treatment”* (ID 15).

Some patients used telemedicine to communicate to the nurse their loneliness: *“There were some older people who called and said they were at home and not going out. They said they were suffering from loneliness. Family or neighbours did the shopping, but the older persons were terrified of the pandemic*” (ID 07).

#### 3.5.3 Difficult interpersonal contact with older patients during tele-treatments

Nurses acknowledged that various difficulties the patients had related to the processes of senile change, which made it difficult in this situation to use the required medical assistance: *“Those older patients do not always know how to communicate their condition, or their condition has simply deteriorated to such an extent that the family has had to become more involved in their care”* (ID 12).

They found it difficult to understand and accept new solutions such as e-prescriptions or e-referral: *“Patients did not understand the situation because, you know, these are older generation people. For example, it was difficult for them to get used to something like e-prescription or e-referral. They could not adjust and often came to the clinic in person. It was known that we only greeted them at the door. But it was hard for these older people to understand this situation and to get used to it*” (ID 15).

In addition, the participants perceived great difficulties that occurred during the tele-treatment of older patients. These difficulties were often due to the health problems characteristic of older people: *“Sometimes it happened that the family or carers called us and contacted us by phone because the patient had a hearing loss or was visually impaired or did not know how to use the phone”* (ID 18).

### 3.6 Theme 4: PHC nurse support and positive change after the COVID pandemic experience

This theme highlights the importance of workplace support and personal support networks for nurses and the positive changes associated with the COVID pandemic experience in PHCs.

#### 3.6.1 Information support

Dynamically evolving health situations during the pandemic required healthcare facilities including PHCs, to act quickly to meet the epidemic requirements, often governed by supreme regulations and orders. Nurses received information support, which they reported as follows: *“Then came regulations, executive orders, how to deal with vaccinations, or with patients”* (ID 04) and: “*There was also information support, because if any new procedures came in, the management let us know”* (ID 14). However, some nurses expressed an overabundance in the amount of information provided, which made it difficult to work: *“There was all sorts of information, but it was all so dynamic that sometimes you got lost in it, and instead of being a support it confused you even more”* (ID 15).

#### 3.6.2 Material support

Almost all nurses expressed positive opinions about the factual and material support related to the personal protective equipment available in the PHC to protect against virus infection: “*As for the management, the employer, they provided us with all personal protective equipment and there was a financial allowance for working during the pandemic*” (ID 01) and: *“As far as all these personal protective equipment was concerned, we basically had access to masks, to gloves, to aprons, to visors, to everything. We did not have to worry about that. That was always provided, and there was never a shortage of that*” (ID 02).

#### 3.6.3 Emotional support

Participants highlighted the support received from colleagues, family, friends and neighbors, and patients as their main source of support. The following comments from nurses confirmed these experiences: *“As a nursing team, we are very supportive of each other, for example, in the fact that one of us always suggests something, we always solve difficult situations in the PHC together. The nursing team was also a big support in the pandemic”* (ID 05) and: *“And the other support was possibly that mental one, but more from workmates. We shared our concerns and it gave us a bit of encouragement”* (ID 13), and also: *“From patients, from doctors, from colleagues too, we supported each other during this difficult time”* (ID 08) and: *“The biggest support I got was from my family. More so in the form of conversation. From friends too. Some were happy that I was able to work during those times, because it was a difficult time though*” (ID 01).

However, there were situations where such support was lacking and ended in the nurse leaving the profession: *“It was a late decision for me to leave this clinic because I became depressed after almost two years of working remotely and on the phone through the pandemic. I did not receive any support”* (ID 09).

#### 3.6.4 Nurses’ educational needs

Nurses reported deficits in meeting key learning needs related to safety when caring for patients with COVID-19, including donning and doffing PPE and performing nursing procedures, disinfection or taking samples for testing: “*I mean, in our PHC, there was no additional training*” (ID 02) and: “*We tried, but we did not have such simple, clear guidelines like where to change, what to do with our clothes, which was the biggest problem*” (ID 10).

#### 3.6.5 Positive changes in the PHC after the COVID pandemic experience

Despite the many challenging experiences brought about by the new care situations that arose during the COVID pandemic, nurses pointed to positive experiences which brought about changes in the PHC care system, which focused on seeing the value of tele-treatment in care: *“The introduction of telemedicine. If a patient is afraid to leave the house and wants to contact the doctor, they now have the possibility to do so, or even to get a prescription; you do not have to go out on purpose and expose yourself; you just have to call”* (ID 13).

In addition, the nurses saw a positive adherence to hygiene procedures and not neglecting the symptoms of infection: *“People have started to observe hygiene more. For example, when they have symptoms of an infection, they tend not to go to work, but try to do a check-up first, to make sure that everything is ok, and that they do not infect others. They are more careful about their health, but also about the other person’s health”* (ID 12).

They also noticed a positive approach to COVID vaccination among older patients: *“As for the older persons they were keen to be vaccinated against COVID, very keen, and even took the third dose. They always emphasized that ‘I am vaccinated, please do not be afraid of me’; something like that was said as a joke”* (ID 06).

Difficult experiences also prompted reflections of joy in life despite difficulties: *“On a positive note, it’s important to enjoy every day because we do not know what tomorrow will bring us”* (ID 05).

## 4 Discussion

Our study investigated the experiences of nurses in Poland who cared for older patients in a primary care setting over the five years of the COVID pandemic. In accordance with the scoping review, this study is one of the limited studies in Poland evaluating PHC nurses’ experiences of caring for older patients during a long period that included five waves of the pandemic. Other studies related to the care of COVID-19 patients have mainly focused on nurses’ experiences in hospital-based specialist care (17–19, 28) or the mental state and well-being of healthcare professionals (29–31), as well as the experiences of Primary Healthcare Nurses during the COVID-19 pandemic (32–35).

The findings of this study allowed for the division into four main themes and seventeen subthemes that covered a wide range of topics related to PHC work during the COVID-19 pandemic, including the physical and psychological suffering associated with the COVID-19, the demands and challenges in the PHC work, the difficulties of caring for older patients, and positive changes resulting from the experience gained during the pandemic. The findings of other researchers (36, 37) are also consistent with the results of our study, which showed that nurses attempted to provide physical, mental and emotional care for COVID-19 patients as befits their professional status.

### 4.1 Impact of the COVID-19 pandemic on the physical and mental health of PHC nurses

Our study was conducted after the fifth wave of the pandemic in Poland, with nurses having experience working in PHCs from the first wave stage of COVID-19. Despite their acquired experience during the pandemic’s successive waves, nurses reported physical and psychological suffering, being overburdened with work, being obliged to prevent infection under adverse conditions and having close contact with confirmed COVID-19 cases. During the COVID-19 pandemic, PHC nurses faced psychological and physical distress due to their experiences with fear, uncertainty about their family members’ health and their patients’ health conditions, as well as social stigma. As the pandemic progressed, their perspective on these experiences evolved. An in-depth analysis by Cho and Kim (38) aiming at categorizing and characterizing the psychological reactions of nurses, who experienced the care of a patient with COVID-19, was based on a methodology which showed that psychological reactions can be divided into three areas: (1) fear of social stigma, (2) anxiety related to the risk of infection and (3) the burden of infection prevention and nursing control. In our findings, all of these areas were reported by nurses through their experience of caring for older patients in the PHC.

The impact that the COVID pandemic-19 had on the physical and mental health of the nurses was also identified in a systematic review of 18 selected studies (39). The results of this review indicated that the main psychological impact on nurses during the COVID pandemic-19 was related to fear, anxiety, stress and depression. Fear of infecting family members or of infecting one’s elf while working was the main influence perceived by nurses in the above review. The results of the qualitative analysis in our study are consistent with the results of the above systematic review (39) and results of the studies by Eghbali et al. (40); Blake et al. (41), Kackin et al. (17).

### 4.2 Primary health care experience with the COVID-19 pandemic

Primary care experiences with the COVID pandemic-19 around the world were similar in terms of stress levels among medical staff. They involved the need for rapid technological adaptation and changes in care delivery strategies, which could often be incurred at the expense of routine care (42). In order to prevent virus transmission, the PHC facility work was reorganized during the pandemic. This included limiting direct medical care in favor of telephone medial consultations and enhancing facility restrictions. In our survey results, the nurses reported the factor of PHC work reorganization as one of the main factors in changing the current work system. In addition, in the experiences of our PHC nurses during the long period of the COVID pandemic, themes such as reduced accessibility to medical care, fewer face-to-face clinic appointments in the PHC, tele-treatment, ongoing interventions related to COVID-19 disease in the PHC and restrictive protective measures against transmission of COVID-19 infection were reported.

The restriction of PHC appointments during the COVID-19 pandemic was a common practice. Significant reductions in the use of direct outpatient services during peak infections were reported in many countries. A United States study found that visits to ambulatory care practices fell by almost 60% in March 2020 and around 50% for primary care visits over the same period (43). The decline in in-person appointments in primary care during the pandemic was a global phenomenon in many other countries (44).

#### 4.2.1 PHC challenges in caring for geriatric patients during the COVID-19 pandemic

Maintaining access to healthcare during an outbreak requires balancing primary care services and protecting vulnerable populations from COVID-19 infections in older patients, among others. PHC facilities faced numerous difficulties when providing care for older patients during the pandemic. Older patients were disoriented, isolated and reluctant to receive medical care. There were also several reports of late and severe reports of lack of COVID-19 treatment and care, presumably due to the limited access to care or fear of nosocomial infection in healthcare facilities (45–47). Interruptions in routine care during the pandemic have also been described in other studies (48, 49), which highlighted the need to strengthen PHCs’ capacity to provide access to a wider range of regular health services during the pandemic and to reduce the potential harmful effects associated with delayed care for patients with chronic diseases and older patients. This theme has also been perceived as important and reported by the nurses surveyed in our research.

Reducing the routine treatment of older patients with chronic diseases could have important and unpredictable adverse health consequences. Abandonment of health care is associated with poorer health outcomes, including complications and hospitalization (50). Therefore, older people must have access to healthcare services during the pandemic, both in emergency and PHC capacities. In our study, PHC nurses have highlighted the emerging difficulties in caring for older patients during the COVID-19 pandemic. These were mainly related to older people’s concerns about the COVID-19 infection during visits and focused on COVID-19-related health problems among older patients rather than seeing other disease problems. Nurses also reported problems related to impaired interpersonal contact with older patients during tele-treatments. Observed perceptions of the needs and resources of patients in the responses of participants in this study also reflected concerns about the ‘digital gap’, which continues to be a significant barrier for older people in using telemedicine technology and its use required the assistance of carers (51). In the experience of geriatric care workers using telemedicine to care for older patients in response to the COVID-19 pandemic, the findings suggest that telemedicine can greatly facilitate much of the geriatric care that is traditionally provided in person, while being less useful in providing certain aspects of geriatric care to older patients, their carers and families (6).

In addition, according to our reports, PHC nurses have observed some positive changes as a result of the pandemic, despite the numerous occupational challenges. These changes include widespread use of online and telephone medical consultations, adherence to hygiene procedures, not underestimating infections, and an increased interest in vaccination. Kheirandish et al. (52) have identified additional aspects of change and coping with challenges, such as providing comprehensive patient care, meeting safety challenges, fulfilling professional duties, and delivering high-quality health care to COVID-19 patients (15, 16, 53).

### 4.3 Strengths and limitations

This research is one of the studies addressing the actual mindset of PHC nurses providing geriatric care who were participating in the reorganization of the PHC system during the COVID-19 pandemic. By relating the thinking of nurses in the first year and a half of the COVID-19 pandemic, the results of our study provided additional unique insights into the thoughts and feelings of PHC nurses in geriatric care that can be applied in other crisis situations. The structure of our study included experiences over a prolong period of the pandemic, and the results showed that PHC nurses continuously evaluate their own encounters and professional activities, which shapes their knowledge and beliefs about how to use them in practice. As such, the findings presented here should provide a snapshot of the current needs of PHC nurses in caring for older patients in outpatient and home settings, which are likely to evolve as we continue to take advantage of the opportunity created during the systemic transformation period during the COVID-19 pandemic to deliver geriatric care in PHC.

The study has several limitations. Firstly, the findings are limited to the experience of PHC nurses caring for older patients in the eastern region of Poland. Data collected in qualitative research during the pandemic may not take into account the distinctiveness of situational interactions in other crises. Moreover, the recollections of other PHC team members, i.e., PHC doctors, geriatricians or non-medical staff, who are also integral supporting members in some PHC teams, were not taken into account. The study also failed to include the patients’ and carers’ thoughts related to the subject at hand. Another limitation is in the use of a single research tool, such as interviews. Therefore, future research should consider applying related tools, such as questionnaires, which can be useful to investigate broader insights.

## 5 Conclusion

This exploratory qualitative study provides insight into the thinking of PHC nurses who cared for older patients during the COVID-19 pandemic in Poland. The results provide a perspective on the difficulties and dynamics of change in the nurses’ work system throughout the year and a half of the pandemic in the context of implementing telemedicine as a method of providing appointments and the new challenges faced by nurses. On the other hand, they present nurses’ sensitivity to the health and the emotional and social needs of older patients receiving PHC advice. This experience helps to understand that difficult and unusual situations emphasize the need for a subjective and holistic approach to the problems of geriatric patients in the PHC service system. The research uncovers the weaknesses of the PHC system and shows aspects that need to be worked on to strengthen the efficiency and quality of geriatric care.

## Data availability statement

The data analyzed in this study is subject to the following licenses/restrictions: the data presented in this study are available on reasonable request from the corresponding author. Requests to access these datasets should be directed to BS, email: barbaraslusarska@umlub.pl.

## Ethics statement

The studies involving humans were approved by the Bioethics Committee of the Medical University of Lublin (Number: KE-0254/73/2021). The studies were conducted in accordance with the local legislation and institutional requirements. The participants provided their written informed consent to participate in this study.

## Author contributions

BŚ: Writing – review & editing, Writing – original draft, Validation, Supervision, Resources, Project administration, Methodology, Investigation, Formal analysis, Data curation, Conceptualization. GN: Writing – review & editing, Visualization, Software, Resources, Investigation, Formal analysis. AC-R: Writing – review & editing, Writing – original draft, Visualization, Supervision, Resources, Project administration, Methodology, Investigation, Formal analysis, Data curation, Conceptualization. LM: Writing – review & editing, Supervision, Resources, Project administration, Methodology, Investigation.
